# Enhanced photoinduced mass migration in supramolecular azopolymers by H-bond driven positional constraint†

**DOI:** 10.1039/d1tc02266k

**Published:** 2021-08-06

**Authors:** Fabio Borbone, Stefano Luigi Oscurato, Salvatore Del Sorbo, Filippo Pota, Marcella Salvatore, Francesco Reda, Pasqualino Maddalena, Roberto Centore, Antonio Ambrosio

**Affiliations:** Department of Chemical Sciences, University of Napoli Federico II, Complesso Universitario di Monte Sant’Angelo, Via Cintia Naples 80126 Italy fabio.borbone@unina.it; CNST@POLIMI – Fondazione Istituto Italiano di Tecnologia, Via Pascoli 70 Milan 20133 Italy antonio.ambrosio@iit.it; Department of Physics E. Pancini, University of Napoli Federico II, Complesso Universitario di Monte Sant’Angelo, Via Cintia Naples 80126 Italy

## Abstract

Here we investigated the role of hydrogen bonding in the design of supramolecular azopolymers with a highly directional and constrained azobenzene–chain interaction involving the aromatic ring of the photoactive molecule, by exploiting the 2-aminopyrimidine/carboxylic acid supramolecular synthon as the tool for molecular recognition. We have shown that this approach is advantageous for producing affordable and versatile photopatternable azomaterials by complexation with polyacrylic acid (PAA). Molecular model complexes were successfully prepared and characterized by X-ray diffraction analysis and FTIR spectroscopy to reveal the multiple, non-ionic interaction occurring between the azobenzene units and the polymer chains. Surface photopatterning of thin films, driven by the typical mass migration phenomenon occurring in azopolymers, resulted strongly enhanced with increasing azobenzene content until equimolar composition. Results show that polymers with synthon-based azobenzenes markedly outperform single H-bonded systems bearing azomolecules with similar structure and electronic properties. We finally demonstrated that the azobenzene units can be easily extracted from a photopatterned film by a simple solvent rinse and without any chemical pre-treatment, leaving the periodicity of the inscribed surface relief gratings unaltered. This result was enabled by the orthogonal solubility of the components in the supramolecular system.

## Abstract 2

SRG inscription on azopolymers films is strongly enhanced by constrained azobenzene–chain interaction established through double H-bond supramolecular synthon. Selective removal of azobenzene from patterned samples is possible by solvent rinse.

## Graphical abstract

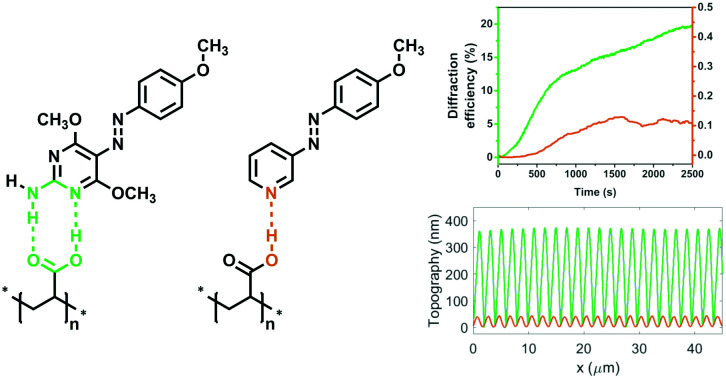

## Introduction

Materials functionalized with azobenzene or its derivatives have been the object of extended research for decades because of their ability to respond to light.^1^ The absorption of radiation with proper wavelength gives rise to photoisomerization of the azomolecules between *trans* and *cis* isomers showing a strong conformational difference. The possibility of sustaining the continuous *trans*–*cis*–*trans* cycle through irradiation is at the origin of the reorientation of the azomolecules under illumination with a linearly polarized laser beam. The resulting induced anisotropy and birefringence can be advantageously exploited to drive the orientation of mesophases in liquid crystals based materials^2–5^ or in optical devices for reversible holographic data storage.^6–9^ Macroscopic mass migration occurring on the surface of azopolymers thin films is recognized as another important phenomenon resulting from *trans*–*cis* photoisomerization in azomolecules.^10^ Irradiation of the surface with an interference light pattern of polarized laser beams can induce highly directional polarization dependent mass transport, with formation of topographic modulations known as surface relief gratings (SRG), which replicate the illuminating intensity pattern^11^ and can be erased by irradiation with circularly polarized light or by heating.^12^ The result is a single-step all-optical reversible process to control surface topography which can be exploited in numerous technological applications, from photonics and optoelectronics such as DFB lasers^13,14^ and organic solar cells^15^ to complex textured surfaces,^16,17^ templates for lithography^18^ or tuning of wettability.^19^ In a polymeric material, mass migration and SRG formation efficiently occur when azochromophores are linked to the polymer chain, thus mainly covalently bonded side-chain and main-chain polymer systems were initially investigated. However, in the last decades the supramolecular approach has emerged as a powerful tool in the design of azomaterials, because of the high flexibility offered by simple synthetic procedures, mainly based on mixing components in solution, the possibility to investigate a wide range of noncovalent interactions of variable strength and the effortless optimization of composition. Indeed, different types of supramolecular chromophore–chain interactions have been successfully exploited to produce efficient SRG formation, from non-directional and strong ionic bonds to hydrogen and halogen bonding.^20–27^ Supramolecular polymers based on halogen-bonded azochromophores grafted onto poly-4-vinylpyridine have revealed interesting performances in terms of SRG inscription efficiency and in some cases outperformed hydrogen-bonded systems based on the same photopassive polymer, although they suffered from phase separation at high azochromophore content.^28^ The reason of the higher efficiency has been ascribed to the high directionality of the halogen bond, in which the σ-hole on the aromatic halogen involved in the electrostatic interaction is confined on the elongation of the R–X covalent bond axis, resulting in a more rigid chromophore–chain connection, improved “dragging” of the chains by the photoactive azomolecules during isomerizations and hence better overall mass migration compared to conventional H bonded systems. Moreover, given the same directionality, efficiency increased with halogen bond strength.^29^ Herein we propose a model of a supramolecular azopolymer in which the degrees of freedom in the relative motion of azobenzene unit and polymer chain are further reduced through the direct involvement of the azobenzene aromatic ring in an additional weak bond. We investigated the role of hydrogen bonding in increasing strength and directionality of the chromophore–chain interaction and the effect on the efficiency of SRG formation, by exploiting the action of cooperative multiple hydrogen bonds. In this regard, we used the concept of the supramolecular synthon as a design element to provide a strong molecular recognition system for a stable and directional grafting. The double hydrogen bond occurring between a carboxylic acid group and a 2-aminopyridine type moiety is recognized as a robust supramolecular heterosynthon in crystal engineering and proved as a useful tool to produce stable amorphous solid dispersions.^30,31^ In this work, we designed new supramolecular azopolymers by grafting 2-aminopyrimidine functionalized azochromophores onto polyacrylic acid (PAA) and investigated the efficiency of SRG formation.^32^ A comparative study of SRG inscription performances with a single H bonded system revealed a very strong influence of the supramolecular synthon on the efficiency of mass migration.

## Results and discussion

### Synthesis and structural characterization

**Fig. 1:**
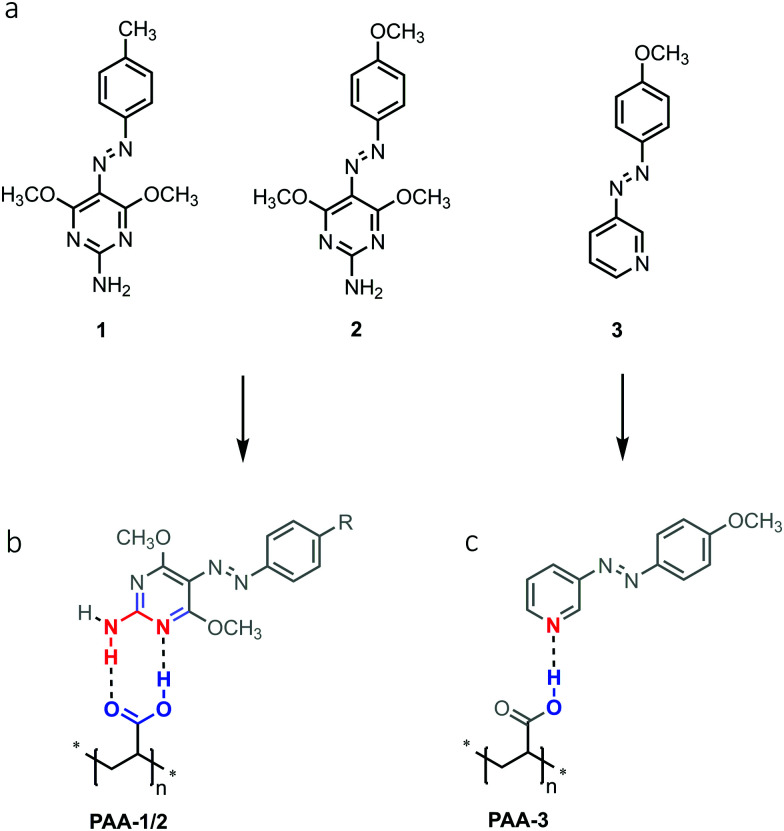
Chemical structure of azochromophores (a) and grafted polymers (b and c).

The azochromophores **1** and **2** reported in Fig. 1 were synthesized in high yields and purity through a simplified single step procedure (see Schemes S1 and S2, ESI†). In brief, diazonium salts (isolated as tetrafluoroborates) were directly coupled onto 2-amino-4,6-dimethoxypyrimidine in dichloroethane, to give the corresponding Azo·HBF_4_ product, then deprotonated and crystallized by treatment with triethylamine in ethanol/water. These compounds were suitable for grafting onto polyacrylic acid (PAA) through complexation based on the supramolecular heterosynthon showed in Fig. 1b. It has been recently demonstrated that this polymer is able to produce very stable amorphous solid dispersions of drugs containing a 2-aminopyridine based pharmacophore. A second type of PAA based polymer was also prepared by using compound **3**, with the aim to compare the mass migration performances of synthon-based polymers with those of a similar system characterized by a single chromophore–chain hydrogen bond (Fig. 1c). To investigate at a molecular level the guest–host interactions occurring in the **PAA-1/2** azopolymers we first studied the crystal structure of the azopyrimidine derivatives and that of model compounds of the azopyrimidine–polymer system by single crystal X-ray diffraction. Compound **1** was crystallized in two polymorphs, named **1-I** and **1-II**, while **2** as one phase. The crystal packing of both the polymorphs of **1** and of compound **2** is driven by the common motif of double hydrogen bonds between the NH_2_ donor and two OCH_3_ acceptors, with no involvement of the aromatic nitrogen of the pyrimidine ring as possible acceptor (Fig. S1–S3, ESI†). This pattern of interactions changes when carboxylic monomers were used to induce molecular recognition. Compound **1** was successfully co-crystallized with methacrylic acid (**MA-1**) to obtain a monomeric model compound of the **PAA-1** polymer. A dimeric model compound of the polymer was also obtained by co-crystallization with bifunctional adipic acid (**AA-1**). Single crystals were grown by evaporation from chloroform/heptane in both cases with high yields. X-Ray diffraction analysis revealed a 1 : 1 ratio for the complex **MA-1**. The azomolecule is in almost planar conformation, and there is no disorder in the azo group. A very stable R^2^_2_(8) cyclic structure with eight atoms and two hydrogen bonds is formed. This structure, in principle, can be realized in three ways: between two carboxylic acid groups (AA homosynthon), between two aminopyrimidine groups (PP homosynthon) and between an acid and an aminopyrimidine group (AP heterosynthon).

**Fig. 2:**
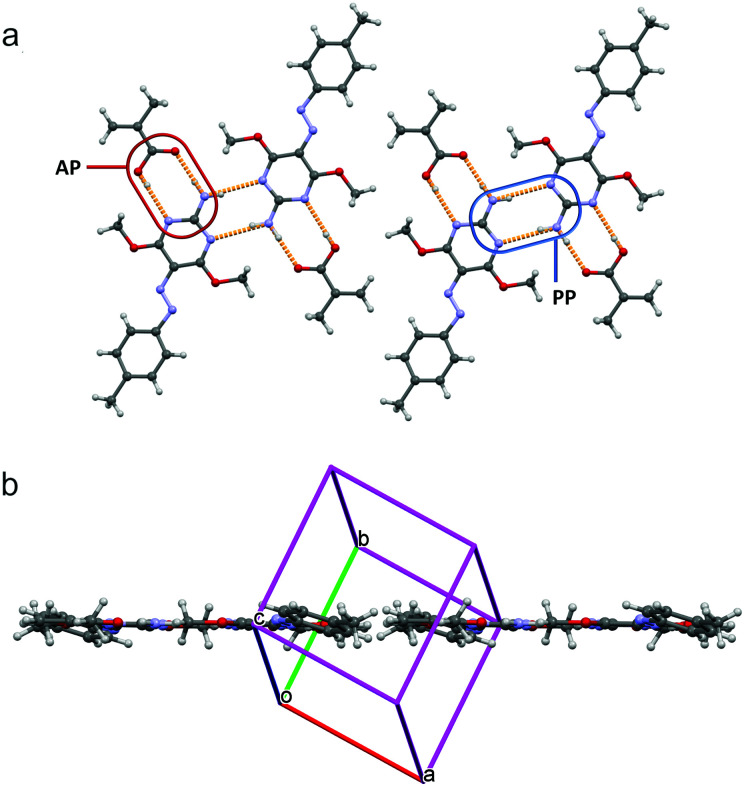
Partial packing of **MA-1**. (a) Face view with H bond pattern in orange; (b) edge view.

**Fig. 3:**
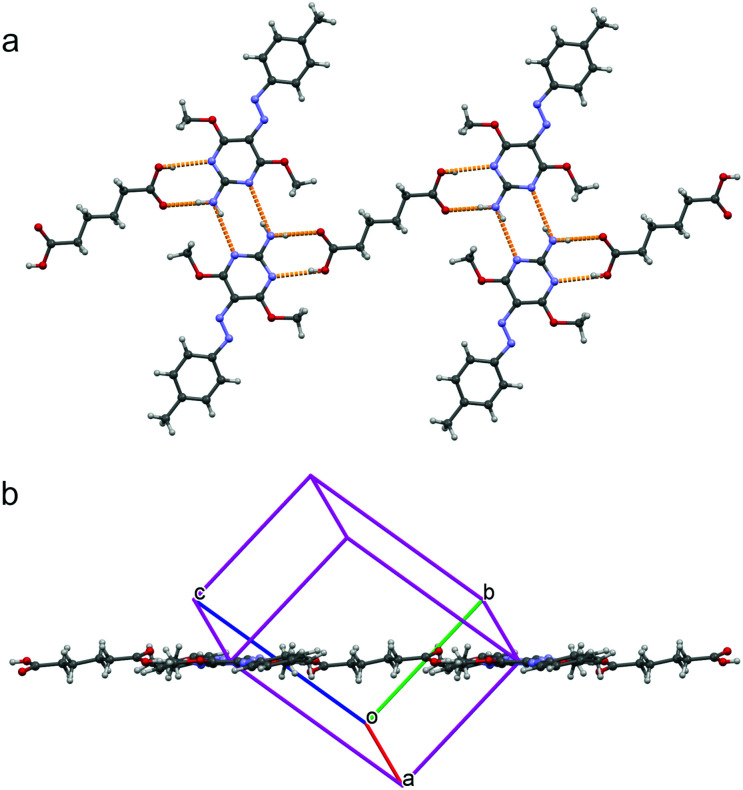
Partial packing of **AA-1**. (a) Face view with H bond pattern in orange; (b) edge view.

The azomolecule in **MA-1** is involved in two double hydrogen bonding patterns (Fig. 2), an AP heterosynthon and a PP homosynthon, and two R^2^_2_(8) ring patterns are formed. In the PP homosynthon, the N–H⋯N hydrogen bond shows N⋯N and H⋯N distances of 3.172(2) Å and 2.32(2) Å respectively, while the bond angle is 168(2)°. In the AP heterosynthon, for the N–H⋯O bond, N⋯O and H⋯O distances are 2.836(2) Å and 1.91(2) Å respectively, while the angle is 175(2)°; for the OH⋯N bond, O⋯N and H⋯N distances are 3.051(2) Å and 2.19(2) Å respectively, with an angle of 175(2)°. The resulting (**MA-1**)_2_ dimers are laterally packed to form infinite layers. These layers are parallel to the lattice planes of Miller indices 1̄21 and stacked with a spacing of 3.28 Å. Crystals of the **AA-1** complex revealed a melting peak at 138 °C in the DSC diagram, which is lower than those of the two components (152 °C for both adipic acid and **1**). The complex **AA-1** crystallized in a 1 : 2 ratio, with each adipic acid molecule involved in two AP heterosynthons with two azomolecules (Fig. 3). Each of these two azomolecules are linked through a PP homosynthon to the azomolecule of another **AA-1** complex, giving rise to a 1-D polymeric chain through alternated PP and AP synthons. These chains are packed to form layers as in **MA-1**, which are stacked with a spacing of 3.38 Å. In the PP homosynthon, the N–H⋯N hydrogen bond shows N⋯N and H⋯N distances of 3.207(2) Å and 2.41(2) Å respectively, while the bond angle is 167(2)°. In the AP heterosynthon, for the N–H⋯O bond, N⋯O and H⋯O distances are 2.895(2) Å and 2.04(3) Å respectively, while the angle is 174(2)°; for the OH⋯N bond, O⋯N and H⋯N distances are 2.974(2) Å and 2.18(2) Å respectively, with an angle of 170(2)°.

The corresponding bond distances in the two complexes are similar and show that the hydrogen bonds in the heterosynthons are shorter, as expected due to the higher acid–base character of this interaction. However, the lengths of C=O and C–O bonds in the carboxylic group (1.198 Å and 1.334 Å respectively for **AA-1**, 1.209 Å and 1.333 Å respectively for **MA-1**) are in the normal range for fully double and single bond respectively and suggest that no proton transfer occurs from hydroxyl to the nitrogen of pyrimidine ring. To support this observation, we performed a statistical analysis of all the compounds containing the same 2-aminopyrimidine/carboxylic acid heterosynthon reported in the CSD database. The mean values of C=O and C–O bond lengths of the 143 hits reported are 1.2127 Å (*σ* = 0.0172) and 1.3084 Å (*σ* = 0.0136) respectively and confirm that formation of carboxylate ion is not expected in this type of heterosynthon. The X-ray analysis confirmed the robustness and non-ionic character of the supramolecular synthon and supported the assignment and comparison of FT-IR signals (*vide infra*).

### Spectroscopic characterization

**Fig. 4:**
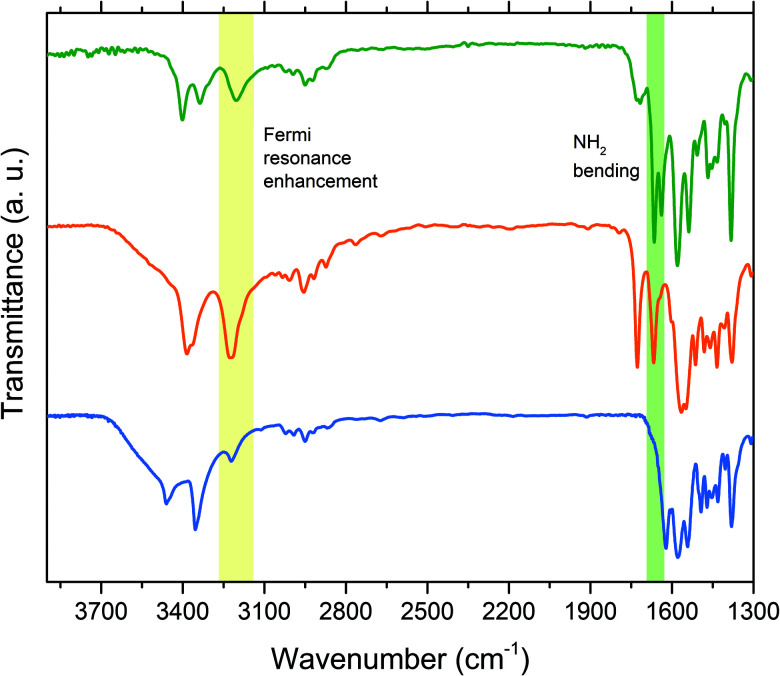
FT-IR spectra of **PAA-11.0** (green line), **AA-1** (orange line) and **1** (blue line).

The azochromophores **1** and **2** were used to prepare the PAA based supramolecular polymers **PAA-1x** and **PAA-2x** with *x* = 0.5, 0.75 and 1.0 dye/polymer molar ratio, by dissolving the proper amount of azochromophore and PAA (*M*_w_ 1800) in dimethylformamide (DMF), in order to have a 1 : 3 azopolymer/solvent weight ratio. The solutions were spin-coated on glass slides or KBr pellets for characterization. All the solutions gave rise to perfectly transparent and amorphous films, without any evidence of phase separation or dye aggregation, even for the highest chromophore content, corresponding to 79.1 wt% and 80.1 wt% respectively for **PAA-11.0** and **PAA-21.0**. The UV-vis absorption spectra of polymers in DMF solution and thin films show maxima at 359 nm and 364 nm for respectively **PAA-1x** and **PAA-2x**, corresponding to the π → π* transition, with a shoulder at longer wavelengths corresponding to the n → π* transition (Fig. S4, S5 and Table S3, ESI†). The spectra of thin films are similar to those in solution and exhibit an expected slight broadening, indicating that no aggregation occurs in the films even at the highest concentration. The relative slight increase of the visible band with decreasing azobenzene concentration observed in both polymer series can be attributed to the absorption by the hydrazone tautomer produced after the partial protonation of the azo groups by the increasing free carboxylic acid groups.^33,34^ The same effect was observed in the UV-vis spectra of **1** and **2** in ethyl acetate added with acetic acid (Fig. S6 and S7, ESI†), while in acetic acid the chromophores resulted completely protonated. The refractive index dispersion of polymers was evaluated by ellipsometry (Fig. S8, ESI†). Values of *n* at the relevant wavelength of 633 nm ranged from 1.638 of **PAA-31.0** to 1.706 of **PAA-21.0** (Table S3, ESI†). In order to investigate the supramolecular interaction in the synthesized azopolymers, a comparative FT-IR analysis was performed on azochromophores, model compounds and polymers. In the IR spectrum of pure PAA (Fig. S9, ESI†), absorption at 1706 cm^−1^ is attributed to the carbonyl stretching of carboxylic acid dimer in the polymer matrix.^31^ This signal is shifted to 1725 cm^−1^ in the grafted azomaterials (Fig. 4 and Fig. S9, S10, ESI†), indicating that the carboxylic acid dimer group is no longer present. The same band is observed at 1727 cm^−1^ in the spectrum of the **AA-1** complex (Fig. 4). Therefore, this band has to be assigned to the carbonyl of the carboxylic group involved in the AP heterosynthon observed in the crystal structure. Evidence of the second hydrogen bond involving the amino group can be gathered from the analysis of the characteristic NH stretching and bending frequencies. In the spectra of pure **1**, the band at 1622 cm^−1^ corresponds to the typical scissoring of the NH_2_ group, as known for amines and reported for 2-amino-4,6-dimethoxypyrimidine.^35^ This bending mode is dependent on the hydrogen bonding and occurs at higher vibrational energy with increasing strength and number of hydrogen bonds.^36^ In **AA-1**, this band is considerably shifted to higher frequency (1667 cm^−1^) because the amino group of the azochromophore is involved in two hydrogen bonds of the PP and AP type synthons, as shown by the X-ray analysis. In the spectra of all **PAA-1x** and **PAA-2x** polymers (Fig. S9 and S10, ESI†) this signal is split at 1665 cm^−1^ and 1640 cm^−1^, suggesting that part of the amino groups in the polymer matrix can participate in second hydrogen bond with surrounding acceptors. The second evidence of hydrogen bonding is given by the analysis of vibrational frequencies of the NH stretching. In the spectrum of **1**, asymmetric and symmetric stretching are visible at 3461 cm^−1^ and 3354 cm^−1^ respectively, while the weak band at 3222 cm^−1^ typically results from the Fermi resonance coupling between an overtone of the NH_2_ bending/scissoring at 1600 cm^−1^ and the symmetric N–H stretching mode. When the amino group is involved in hydrogen bonds, the NH stretching shifts to lower frequencies and its oscillator strength increases significantly. This effect and the reduced energy gap with the overtone transition leads to a strong enhancement of the Fermi resonance coupling and to a dramatic increase of the 3200 cm^−1^ band intensity, dependent on the strength of the hydrogen bonds.^37,38^ This coupling is clearly visible in the spectrum of **AA-1**, where the intensity of the band at 3226 cm^−1^ exceeds that of the double band at 3385 cm^−1^. A very similar coupling and band pattern is observed for polymers (Fig. 4 and Fig. S9, S10, ESI†) and confirms that the double hydrogen bond of the AP heterosynthon effectively takes place within the grafted polymers. The formation of the hydrogen bond between PAA and **3** in **PAA-31.0** was verified by following the shift of the stretching of carbonyl from 1706 cm^−1^ to 1716 cm^−1^ and that of the free pyridyl ring (1020 cm^−1^) to higher frequency, with the appearing of a second band at 1040 cm^−1^ (Fig. S11, ESI†).^39^

### Efficiency of SRG formation

**Fig. 5:**
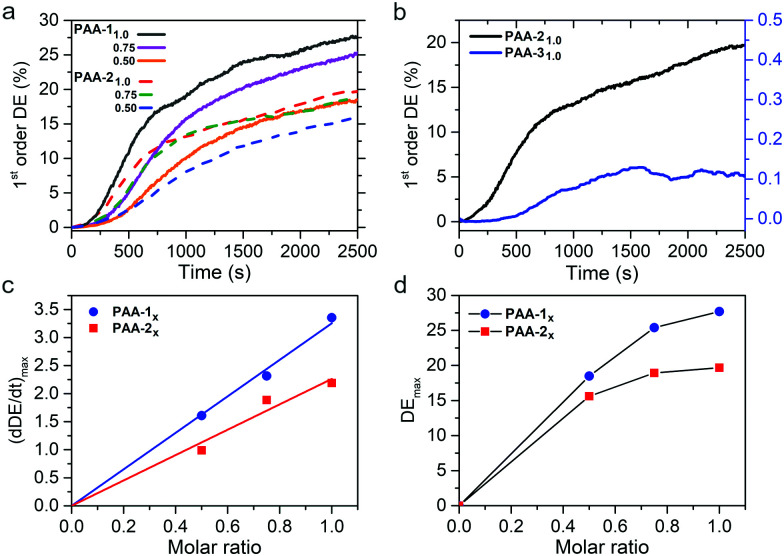
(a) Diffraction efficiency of **PAA-1x** and **PAA-2x** (*x* = 0.5, 0.75, 1.0; film thickness: 880–950 nm; irradiance: 175 mW cm^−2^); (b) diffraction efficiency of **PAA-21.0** and **PAA-31.0**; (c) SRG inscription rate and (d) maximum of diffraction efficiency for **PAA-1x** and **PAA-2x** as a function of composition.

**Fig. 6:**
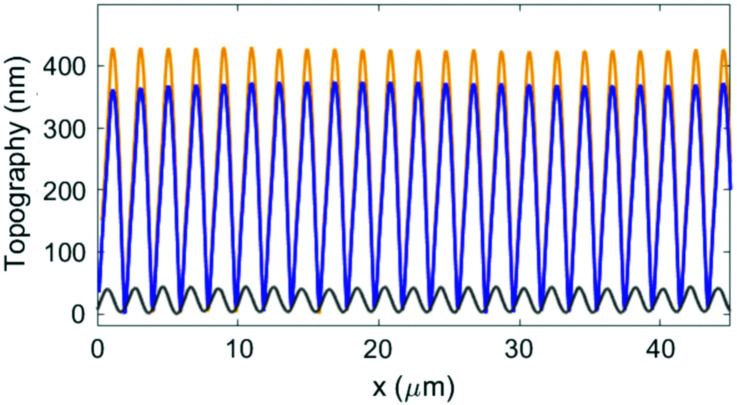
AFM cross-sectional profiles of SRGs inscribed on **PAA-11.0** (orange line), **PAA-21.0** (blue line) and **PAA-31.0** (black line) in the same time lapse.

The efficiency of mass migration was investigated by performing SRG inscription experiments on thin films of the prepared polymers. Structuring was carried out by irradiating the samples with an interference pattern obtained with p-polarized beams, producing regular and high quality SRGs for all polymers. A laser wavelength of 457 nm was used to work in a range of relatively low chromophore absorption, with the aim to compare the performances of synthon-based systems with those of the conventional hydrogen bonded **PAA-31.0** in a regime not affected by thermal effects. Fig. 5a shows the growth of the 1st order diffraction efficiency (DE) produced by the forming grating as a function of time. The efficiency of mass migration can be related to the rate of SRG formation, calculated as the slope of the straight line obtained by fitting the data in the range of linear growth, corresponding approximately to the section of curves between 10% and 30% of the DE maximum value attained at the end of the experiment. These data are reported in Fig. 5c and d for all the polymers as a function of the azobenzene content. The results show that the SRG inscription rate linearly increases with the concentration of the azochromophore in the polymer and a regular growth trend is also observed for the maximum DE value, for both **PAA-1** and **PAA-2**. Interestingly, for each composition **PAA-1** noticeably outperforms **PAA-2** (by 41%, 34% and 18% for *x* = 1.0, 0.75 and 0.50 respectively) although the only structural difference between azochromophores **1** and **2** is the oxygen on the terminal methoxy group of **2**. This electron donor can act as an additional hydrogen bond acceptor within the polymer matrix and contribute to reduce the effectiveness of *cis*–*trans* isomerizations on molecular and chain motions, thus affecting overall mass migration. This different performance is also evident by comparing the AFM cross-sectional profiles of SRGs inscribed on **PAA-1** and **PAA-2** in the same time lapse (Fig. 6, see also Fig. S12 and S13, ESI† for *x* = 0.75 and *x* = 0.50). The modulation depth increased with the azo content for both series, reaching maximum average values of 420 and 355 nm for **PAA-11.0** and **PAA-21.0** respectively. Table S3 (ESI†) reports the corresponding values of DE.

We further compared the SRG formation performances of **PAA-21.0** and **PAA-31.0** at 457 nm. Compounds **2** and **3** show a similar structure and bear the same methoxy group on the unbound ring of the azobenzene structure. Moreover, the same position of the hydrogen bond accepting nitrogen on the pyridyl and pyrimidyl rings is expected to produce the same positioning of the azo unit respect to the polymer chain. The comparison shows that **PAA-21.0** is considerably more efficient than **PAA-31.0** (maximum modulation depth of 40 nm, Fig. 6), although their absorption spectra do not differ significantly (*λ*_max_ of 364 nm and 343 nm for **PAA-21.0** and **PAA-31.0** respectively, Fig. S14, ESI†). Therefore, we evaluated the lifetime of the *cis* isomer for all the **PAA-i1.0** polymers according to a literature procedure (Fig. S15, ESI†).^40^ The *cis*–*trans* thermal isomerization rate of **PAA-31.0** (1.40 ± 0.03 s^−1^) resulted more than three times higher than that of **PAA-11.0** and **PAA-21.0** (0.42 ± 0.03 s^−1^ and 0.38 ± 0.03 s^−1^ respectively), indicating that thermal isomerization of the *cis* isomer is not a limiting factor for the performance of **PAA-31.0**. Conversely, the dramatic efficiency difference suggests that the presence of a second hydrogen bond involving a further position on the aromatic ring of the azobenzene entails, for the same polymer, a considerable increase in the ability of the photoactive units to drag the polymer chains during the structuring of the material. This effect can be reasonably attributed to the nature of the used supramolecular heterosynthon, which is at the same time a weak and reversible but highly directional and constrained interaction, resulting in an improved conversion of energy into effective mass transport.

### Selective removal of azobenzene

**Fig. 7:**
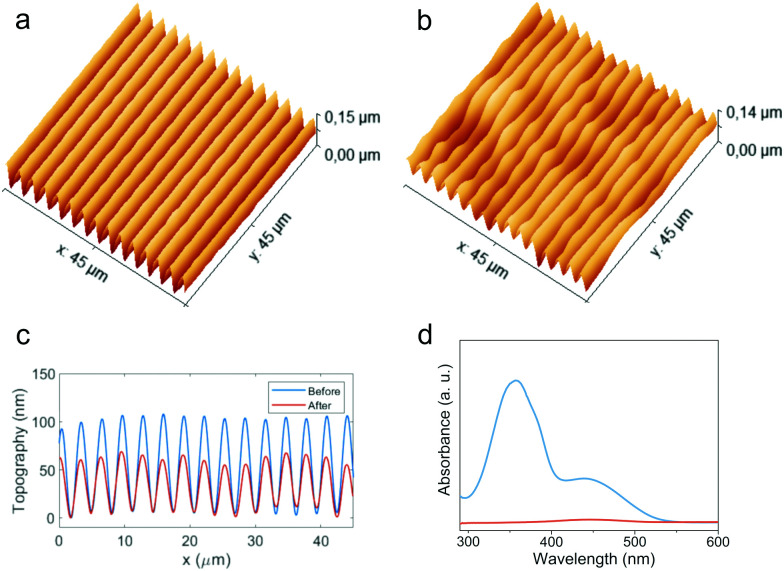
Selective extraction of **1** from SRG of **PAA-10.50**. Topographical AFM image of SRG before (a) and after (b) rinse with solvent; cross-sectional profiles (c) and UV-vis spectra of film (d) before (blue line) and after (orange line) rinse with solvent.

We also investigated the possibility to extract the azobenzene units from the polymer after SRG inscription. This operation is highly desirable in azomaterials for technological purposes, as the removal of the azo units expands the possibility of use in optical devices to the whole range of wavelengths where absorption by chromophores would occur. Seki and coworkers^41^ proposed an H bonding based supramolecular system in which the selective extraction of azobenzene units was successfully performed on a patterned sample after crosslinking of the polymer matrix by exposure to reactive vapors and subsequent rinsing with solvent. Here we demonstrate that this operation could be successfully performed on polymers with *x* = 0.50 without any chemical reaction. Fig. 7 shows AFM topography and UV-vis spectra of a patterned film of **PAA-10.50** before and after rinsing the sample in dichloromethane for 1 min (see Fig. S16 for **PAA-20.50**, ESI†). The film appeared transparent after treatment and the absorption band of the azochromophore disappears in the spectrum (Fig. 7d). However, the profile of inscribed SRG is preserved, although with some loss of quality, accompanied by an expected volume shrinkage, with a visible reduction of the feature heights from about 110 nm to 60 nm (Fig. 7c). This possibility is enabled by the quite different solubility properties of PAA and **1**. While **1** is highly soluble in all common organic solvents, PAA is only soluble in water, alcohols and formamides. Selective extraction of azobenzene units with dichloromethane produces a rapid replacement of the AP type chain–chromophore interactions with the crosslinking AA type dimerization of the freed carboxylic groups, which contribute to the preservation of the SRG dimensional stability. The same procedure applied to samples with higher azo content leads to unsatisfactory results, with severe loss of pattern quality and regularity due to the excessive volume fraction of the material occupied by the azobenzene.

## Experimental

All the reagents were commercially available and used without further purification: *p*-toluidine, 2-amino-4,6-dimethoxypyrimidine, tetrafluoroboric acid 48% aq. sol. (Alfa Aesar), *p*-anisidine, polyacrylic acid average *M*_w_ 1800 (Merck). Optical observations were performed by using a Zeiss Axioscop polarizing microscope equipped with a FP90 Mettler heating stage. ^1^H and ^13^C NMR spectra were recorded on Varian Inova 500 (500 MHz) and Bruker Avance III TM HD (400 MHz) spectrometers. UV-Visible spectra were recorded with a JASCO V-750 spectrometer. The FT-IR measurements were performed on a Thermo Nicolet 5700 FT-IR spectrometer. Solid samples were dispersed in KBr tablets, polymeric materials were cast from solutions. Thin films of azopolymers were prepared by spin coating DMF solutions of the polymers on glass substrates, using a Laurell WS-650Mz-23NPP spin coater.

### X-Ray diffraction analysis

Single crystals of **1-I** and **1-II** suitable for X-ray diffraction analysis were obtained by slow cooling of a heptane solution (or evaporation from acetone) and by recrystallization from ethanol/water, respectively. Single crystals of **2** were obtained by slow evaporation from acetone, while those of the complexes **1-MA** and **1-AA** were grown by evaporation from chloroform/heptane. One selected crystal of each compound was mounted in flowing N_2_ at 173 K on a Bruker-Nonius Kappa CCD diffractometer equipped with Oxford Cryostream apparatus (graphite monochromated MoKα radiation, *λ* = 0.71073 Å, CCD rotation images, thick slices, *φ* and *ω* scans to fill asymmetric unit). Reduction of data and semiempirical absorption correction were done using SADABS program. The structures were solved by direct methods (SIR97 program)^42^ and refined by the full-matrix least-squares method on *F*^2^ using SHELXL-2016 program^43^ with the aid of the program Olex2.^44^ H atoms bonded to C were generated stereochemically and refined by the riding model, those bonded to O and N were found in difference Fourier maps and their coordinates were refined. For all H atoms, *U*_iso_(H) equal to 1.2 *U*_eq_ or 1.5 *U*_eq_ (C_methyl_) of the carrier atom was used.

Crystal data and structure refinement details are reported in Tables S1 and S2 (ESI†). The figures were generated using Mercury CSD 3.3.^45^ All crystal data were deposited at Cambridge Crystallographic Data Centre with assigned number CCDC 2073781 (**1-I**), 2073782 (**1-II**), 2073784 (**1-AA**), 2073785 (**1-AA-RT**), 2073786 (**1-MA**), 2073783 (**2**).†

### Synthesis of **1** and **2**

The tetrafluoroborate diazonium salts of *p*-toluidine (**td**) and *p*-anisidine (**ad**) were synthesized and isolated according to a reported procedure.^46^

**1**. 2-Amino-4,6-dimethoxypyrimidine (1.55 g, 10 mmol) was dissolved in 1,2-dichloroethane (70 ml). **td** (2.93 g, 1.0 equiv.) was added in small portions and reaction stirred overnight at room temperature. The hydrotetrafluoroborate product was deprotonated and purified by recrystallization in ethanol/water in the presence of excess triethylamine, giving large plate-like crystals. Yield: 77%. ^1^H NMR (DMSO-*d*_6_) *δ* 7.52 (d, 2H); 7.32 (s, 2H); 7.27 (d, 2H); 3.91 (s, 6H); 2.35 (s, 3H). ^13^C NMR (DMSO-*d*_6_) *δ* 164.3, 161.2, 151.9, 138.7, 129.5, 121.3, 112.9, 53.9, 20.8.

**2**. The same procedure for the synthesis of **1** was applied using an equimolar amount of **ad** to give **2** in 71% yield. ^1^H NMR (DMSO-*d*_6_) *δ* 7.61 (d, 2H); 7.25 (s, 2H); 7.02 (d, 2H); 3.91 (s, 6H); 3.81 (s, 3H). ^13^C NMR (DMSO-*d*_6_) *δ* 164.2, 161.0, 160.2, 148.0, 122.9, 114.2, 112.8, 55.4, 53.8.

### Synthesis of **3**-(4′-hydroxyphenylazo)pyridine

To a solution of 3-aminopyridine (2.51 g, 26.7 mmol) in H_2_O (11 ml), aqueous HBF_4_ 50 wt% was added (9.1 ml) and the mixture was stirred at 5 °C. A solution of sodium nitrite (1.84 g, 26.7 mmol) in 5 ml water was slowly added, the mixture was stirred at 5 °C for 45 min and then added to a solution of phenol (2.51 g, 26.7 mmol) and NaOH (3.74 g, 93.5 mmol) in 150 ml H_2_O. After 30 min stirring, the pH was lowered to 7 with HCl. The orange precipitate was filtered and washed with water. Yield: 66%. ^1^H NMR (DMSO-*d*_6_) *δ* 10.49 (s, 1H); 9.02 (s, 1H); 8.66 (d, 1H); 8.08 (d, 1H); 7.84 (d, 2H); 7.55 (m, 1H); 6.96 (d, 2H). ^13^C NMR (DMSO-*d*_6_) *δ* 161.6, 151.1, 147.5, 145.9, 145.4, 126.6, 125.2, 124.4, 116.1.

### Synthesis of **3**

3-(4′-Hydroxyphenylazo)pyridine (1.20 g, 6.00 mmol) and K_2_CO_3_ (1.66 g, 12.0 mmol) were added to 30 ml DMF. Dimethyl sulfate (0.757 g, 6.00 mmol) were slowly added and the mixture was stirred at room temperature for 48 h. After addition of water (50 ml) the mixture was extracted with ethyl acetate. The organic phase was washed three times with water, anhydrified with Na_2_SO_4_ and the solvent removed *in vacuo*. Ethanol (10 ml) was added to the liquid residue and a solid precipitated by addition of a solution of sodium acetate (1 g) in 30 ml H_2_O. The brown solid was treated with 600 ml boiling heptane. After cooling, the residue was filtered and the volume was reduced to 20 ml. By cooling the solution, the product was crystallized as an orange solid. Yield: 62%. ^1^H NMR (DMSO-*d*_6_) *δ* 9.07 (m, 1H); 8.71 (m, 1H); 8.16–8.13 (m, 1H); 7.94 (d, 2H); 7.62–7.59 (m, 1H); 7.17 (d, 2H); 3.89 (s, 3H). ^13^C NMR (DMSO-*d*_6_) *δ* 162.5, 151.4, 147.4, 146.2, 146.0, 126.7, 124.9, 124.9, 124.51, 114.7, 55.7.

### SRG inscription

Thin films for SRG inscription experiments were prepared by spin coating filtered 25 wt% DMF solutions of the polymers on glass slides (film thickness 880–950 nm). The samples were kept under vacuum at room temperature for 24 h to remove solvent traces. The interference pattern was set up with two *p* polarized laser beams of 5.5 mW power and 2 mm section each (irradiance 175 mW cm^−2^, wavelength 457 nm, from Samba source of Cobolt 05-01 Series by HÜBNER PHOTONICS) hitting the sample at an incidence angle of 13°40′. The first order diffraction efficiency was monitored with a He–Ne laser beam impinging normally on the growing grating and the diffracted light power was monitored by means of biased photodetectors (DET36A2 model from Thorlabs, based on fast PIN Si photodiodes) placed at the angular directions of the zero and the two first diffraction orders for the 633 nm radiation. The DE was calculated as the power ratio of the He–Ne diffracted beam and the transmitted beam prior to exposure to the 457 nm intensity pattern, by averaging the signal of both −1 and +1 first orders.

## Conclusions

Here we demonstrated the possibility to exploit polyacrylic acid as an affordable polymer matrix to produce supramolecular azopolymers with high performances and versatility, through a molecular recognition approach based on a hydrogen bond supramolecular synthon. These materials can be prepared with the desired chromophore content without undergoing phase separation or chromophore aggregation until equimolar ratio. The proposed system exploits for the first time a double weak interaction acting on two positions of the aromatic ring of the azobenzene unit. This approach promotes a highly directional chromophore–chain interaction, while also preventing any degree of freedom with respect to rotation around the molecular long axis of azobenzene units. This stronger and more rigid constraint resulted in a huge improvement in mass migration and SRG inscription efficiency compared to a single H-bonded system based on the same polymer matrix and positioning of the azomolecule relative to the chain. Rapid and straightforward removal of the light absorbing chromophores from a photopatterned sample was enabled by the orthogonal solubility properties of the polyacrylic acid and the dye component and demonstrated through simple rinsing of the thin film samples with dichloromethane.

## Conflicts of interest

There are no conflicts to declare.

## Supplementary Material

TC-009-D1TC02266K-s001

TC-009-D1TC02266K-s002

## References

[cit1] Natansohn A., Rochon P. (2002). Chem. Rev..

[cit2] O’Neill M., Kelly S. M. (2000). J. Phys. D: Appl. Phys..

[cit3] Corvazier L., Zhao Y. (1999). Macromolecules.

[cit4] Ware T. H., McConney M. E., Wie J. J., Tondiglia V. P., White T. J. (2015). Science.

[cit5] Zeng H., Wani O. M., Wasylczyk P., Kaczmarek R., Priimagi A. (2017). Adv. Mater..

[cit6] Meng X., Natansohn A., Rochon P. (1996). Supramol. Sci..

[cit7] Eich M., Wendorff J., Reck B., Ringsdorf H. (1987). Makromol. Chem., Rapid Commun..

[cit8] Shishido A. (2010). Polym. J..

[cit9] Hvilsted S., Sánchez C., Alcalá R. (2009). J. Mater. Chem..

[cit10] Rochon P., Batalla E., Natansohn A. (1995). Appl. Phys. Lett..

[cit11] Oscurato S. L., Salvatore M., Maddalena P., Ambrosio A. (2018). Nanophotonics.

[cit12] Viswanathan N. K., Kim D. Y., Bian S., Williams J., Liu W., Li L., Samuelson L., Kumar J., Tripathy S. K. (1999). J. Mater. Chem..

[cit13] Goldenberg L. M., Lisinetskii V., Gritsai Y., Stumpe J., Schrader S. (2012). Adv. Mater..

[cit14] Goldenberg L. M., Lisinetskii V., Gritsai Y., Stumpe J., Schrader S. (2012). Opt. Mater. Express.

[cit15] Na S.-I., Kim S.-S., Jo J., Oh S.-H., Kim J., Kim D.-Y. (2008). Adv. Funct. Mater..

[cit16] Oscurato S. L., Salvatore M., Borbone F., Maddalena P., Ambrosio A. (2019). Sci. Rep..

[cit17] Salvatore M., Borbone F., Oscurato S. L. (2020). Adv. Mater. Interfaces.

[cit18] Lee S., Kang H. S., Park J.-K. (2012). Adv. Mater..

[cit19] Oscurato S. L., Borbone F., Maddalena P., Ambrosio A. (2017). ACS Appl. Mater. Interfaces.

[cit20] Zhang Q., Wang X., Barrett C. J., Bazuin C. G. (2009). Chem. Mater..

[cit21] Gao J., He Y., Liu F., Zhang X., Wang Z., Wang X. (2007). Chem. Mater..

[cit22] Kulikovska O., Goldenberg L. M., Stumpe J. (2007). Chem. Mater..

[cit23] Priimagi A., Cavallo G., Forni A., Gorynsztejn-Leben M., Kaivola M., Metrangolo P., Milani R., Shishido A., Pilati T., Resnati G., Terraneo G. (2012). Adv. Funct. Mater..

[cit24] Priimagi A., Lindfors K., Kaivola M., Rochon P. (2009). ACS Appl. Mater. Interfaces.

[cit25] Vapaavuori J., Valtavirta V., Alasaarela T., Mamiya J.-I., Priimagi A., Shishido A., Kaivola M. (2011). J. Mater. Chem..

[cit26] Gao J., He Y., Xu H., Song B., Zhang X., Wang Z., Wang X. (2007). Chem. Mater..

[cit27] Priimagi A., Cattaneo S., Ras R. H. A., Valkama S., Ikkala O., Kauranen M. (2005). Chem. Mater..

[cit28] Stumpel J. E., Saccone M., DIchiarante V., Lehtonen O., Virkki M., Metrangolo P., Priimagi A. (2017). Molecules.

[cit29] Saccone M., Dichiarante V., Forni A., Goulet-Hanssens A., Cavallo G., Vapaavuori J., Terraneo G., Barrett C. J., Resnati G., Metrangolo P., Priimagi A. (2015). J. Mater. Chem. C.

[cit30] Desiraju G. R. (1995). Angew. Chem., Int. Ed. Engl..

[cit31] Duggirala N. K., Li J., Krishna Kumar N. S., Gopinath T., Suryanarayanan R. (2019). Chem. Commun..

[cit32] Čechová L., Kind J., Dračínský M., Filo J., Janeba Z., Thiele C. M., Cigáň M., Procházková E. (2018). J. Org. Chem..

[cit33] Matazo D. R. C., Ando R. A., Borin A. C., Santos P. S. (2008). J. Phys. Chem. A.

[cit34] Dunn N. J., Humphries W. H., Offenbacher A. R., King T. L., Gray J. A. (2009). J. Phys. Chem. A.

[cit35] Sundaraganesan N., Sathesh Kumar K., Meganathan C., Dominic Joshua B. (2006). Spectrochim. Acta, Part A.

[cit36] Huo Q., Dziri L., Desbat B., Russell K. C., Leblanc R. M. (1999). J. Phys. Chem. B.

[cit37] Mishra S., Kuo J. L., Patwari G. N. (2018). Phys. Chem. Chem. Phys..

[cit38] Greve C., Nibbering E. T. J., Fidder H. (2013). J. Phys. Chem. B.

[cit39] Berg E. R., Freeman S. A., Green D. D., Ulness D. J. (2006). J. Phys. Chem. A.

[cit40] Barrett C., Natansohn A., Rochon P. (1995). Chem. Mater..

[cit41] Zettsu N., Ogasawara T., Mizoshita N., Nagano S., Seki T. (2008). Adv. Mater..

[cit42] Altomare A., Burla M. C., Camalli M., Cascarano G. L., Giacovazzo C., Guagliardi A., Moliterni A. G. G., Polidori G., Spagna R. (1999). J. Appl. Crystallogr..

[cit43] Sheldrick G. M. (2015). Acta Crystallogr., Sect. C: Struct. Chem..

[cit44] Dolomanov O. V., Bourhis L. J., Gildea R. J., Howard J. A. K., Puschmann H. (2009). J. Appl. Crystallogr..

[cit45] Macrae C. F., Bruno I. J., Chisholm J. A., Edgington P. R., McCabe P., Pidcock E., Rodriguez-Monge L., Taylor R., van de Streek J., Wood P. A. (2008). J. Appl. Crystallogr..

[cit46] Erb W., Hellal A., Albini M., Rouden J., Blanchet J. (2014). Chem. – Eur. J..

