# Supplementary material for: Enhanced photoinduced mass migration in supramolecular azopolymers by H-bond driven positional constraint
Source: J Mater Chem C Mater. 2021 Aug 6;9(34):11368–75. doi: 10.1039/d1tc02266k (PMC8411878; doi:10.1039/d1tc02266k)
Supplement: TC-009-D1TC02266K-s001 [file TC-009-D1TC02266K-s001.pdf]

Electronic Supplementary Material (ESI) for Journal of Materials Chemistry C.
This journal is © The Royal Society of Chemistry 2021

Supplementary Information

Enhanced photoinduced mass migration in supramolecular azopolymers by H-bond driven positional constraint

Fabio Borbone,*[a,c] Stefano Luigi Oscurato,[b,c] Salvatore Del Sorbo,[c] Filippo Pota,[a] Marcella Salvatore,[b] Francesco Reda,[b] Pasqualino Maddalena,[b,c] Roberto Centore[a] and Antonio Ambrosio*[c]

[a] Department of Chemical Sciences, University of Napoli Federico II, Complesso Universitario di Monte Sant'Angelo, Via Cintia, 80126 Naples, Italy
[b] Department of Physics E. Pancini, University of Napoli Federico II, Complesso Universitario di Monte Sant'Angelo, Via Cintia, 80126 Naples, Italy
[c] CNST@POLIMI - Fondazione Istituto Italiano di Tecnologia, Via Pascoli 70, 20133 - Milano - Italy

*Email: fabio.borbone@unina.it; antonio.ambrosio@iit.it

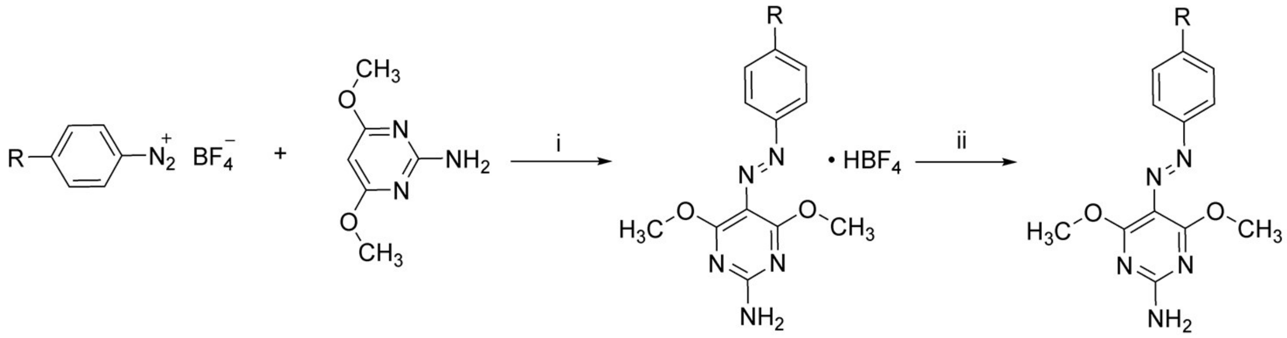

Scheme S1 Synthesis scheme of **1** (R = CH_3) and **2** (R = OCH_3). (i) 1,2-dichloroethane; (ii) triethylamine/ethanol/water.

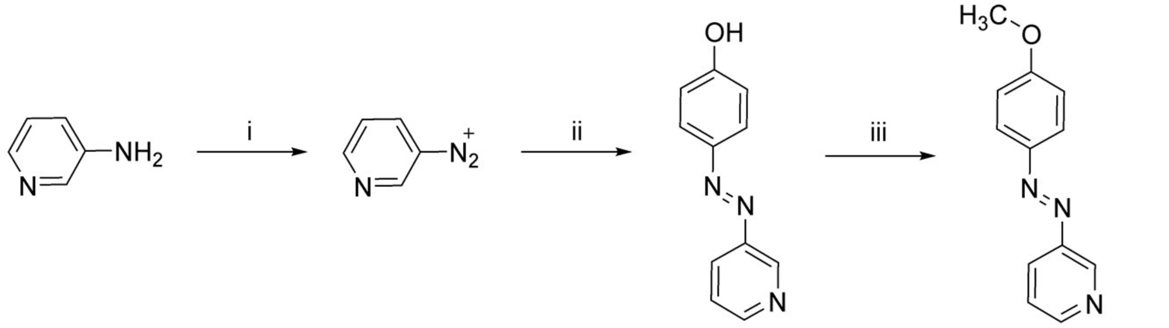

Scheme S2 Synthesis scheme of **3**. (i) $NaNO_2/HBF_4$; (ii) phenol/NaOH; $K_2CO_3/DMF/Me_2SO_4$.

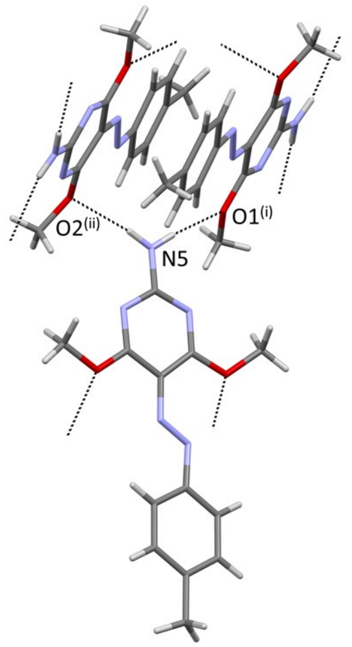

Fig. S1 Hydrogen bonds between NH_2 and OCH_3 in the crystal packing of **1-I**: N5–H···O1$^{(i)}$: 0.85(5), 2.25(5), 3.090(5) Å, 169(3)°, (i)=x, 0.5-y, 0.5+z; N5–H···O2$^{(ii)}$: 0.90(3), 2.33(3), 3.231(3) Å, 173(3)°, (ii)=1-x, 0.5+y, 1.5-z.

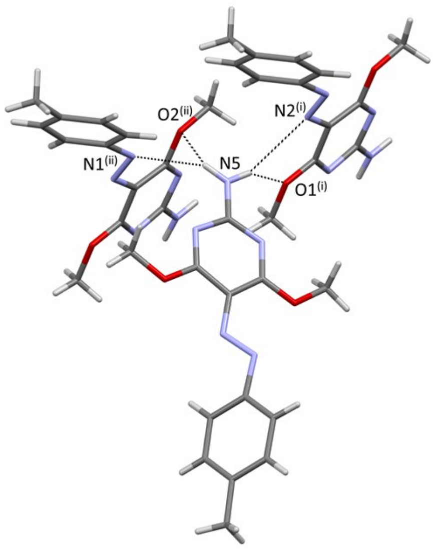

Fig. S2 Strong and weak hydrogen bonds in the crystal packing of **1-II**: N5–H···O1$^{(i)}$: 0.90(2), 2.35(2), 3.131(2) Å, 145(2)°, N5–H···N2$^{(i)}$: 0.90(2), 2.73(2), 3.549(3) Å, 152(2)°, (i)=1-x, 0.5-y, 0.5+z; N5–H···O2$^{(ii)}$: 0.88(2), 2.64(2), 3.139(2) Å, 117(2)°, N5–H···N1$^{(ii)}$: 0.88(2), 2.41(2), 3.211(3) Å, 151(2)°, (ii)=x, 0.5-y, 0.5+z. Only the most populated component of the disordered molecular portion is shown for clarity. Hanging contacts are not shown.

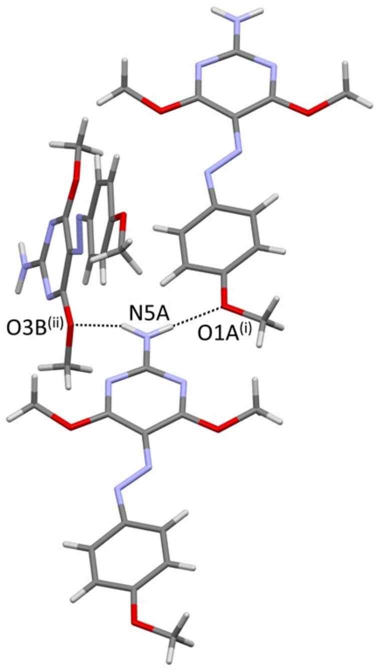

Fig. S3 Hydrogen bonds between NH_2 and OCH_3 in the crystal packing of **2**: N5A–H···O1A$^{(i)}$: 0.86, 2.12, 2.969(2) Å, 169°, (i)=x, 1+y, z; N5A–H···O3B$^{(ii)}$: 0.86, 2.26, 3.039(6) Å, 150°, (ii)=0.5-x, 0.5+y, z. Only the most populated component of the disordered molecule B is shown for clarity. Hanging contacts are not shown.

Table S1 Crystal data and structure refinement details for phase **1-I**, **1-II** and **2**.

	**1-I**	**1-II**	**2**
CCDC number	2073781	2073782	2073783
Empirical formula	$C_{13}H_{15}N_5O_2$	$C_{13}H_{15}N_5O_2$	$C_{13}H_{15}N_5O_3$
Formula weight	273.30	273.30	289.30
T (K)	293(2)	293(2)	293(2)
λ (Å)	0.71073	0.71073	0.71073
Crystal system	Monoclinic	Monoclinic	Orthorombic
Space group	*P2₁/c*	*P2₁/c*	*Pbca*
a (Å)	10.039(4)	6.632(3)	13.7630(16)
b (Å)	13.245(5)	20.892(8)	13.552(4)
c (Å)	14.631(5)	10.929(4)	30.386(9)
a (°)	90	90	90
β (°)	132.51(2)	108.57(2)	90
γ (°)	90	90	90
V (Å^3)	1434.2(10)	1435.4(10)	5667(2)
Z	4	4	16
D_{calc} (Mg/m^3)	1.266	1.265	1.356
μ (mm^{-1})	0.090	0.090	0.100
F(000)	576	576	2432
θrange (°)	2.43 – 27.50	2.77 - 27.50	2.50 – 27.50
Reflections collected / unique	14192/3264	8904/3199	37423/6430
[R(int)]	[0.0399]	[0.0335]	[0.0389]
Data/restraints/parameters	3264/0/191	3199/20/227	6430/153/452
Goodness-of-fit on F^2	1.073	1.050	1.041
Final*R1*, *wR2* indices [I>2*s*(I)]	0.0678, 0.1641	0.0488, 0.1267	0.0535, 0.1249
Final *R1*, *wR2* indices (all data)	0.1097, 0.1989	0.0828, 0.1482	0.0960, 0.1458
Largest diff. peak / hole (eA^{-3})	0.30/-0.29	0.19/-0.19	0.27/-0.27

Table S2 Crystal data and structure refinement details for **1-AA**, **1-AA-RT** and **1-MA**.

	**1-AA**	**1-AA-RT**	**1-MA**
CCDC number	2073784	2073785	2073786
Empirical formula	$C_{16}H_{20}N_5O_4$	$C_{16}H_{20}N_5O_4$	$C_{17}H_{21}N_5O_4$
Formula weight	346.37	346.37	359.39
T (K)	173	293	173
λ (Å)	0.71073	0.71073	0.71073
Crystal system	Triclinic	Triclinic	Triclinic
Space group	*P-1*	*P-1*	*P-1*
a (Å)	8.095(2)	8.1330(10)	6.8530(7)
b (Å)	9.351(2)	9.5050(12)	7.5100(16)
c (Å)	11.6500(14)	11.7310(12)	17.512(3)
a (°)	96.706(14)	95.988(11)	95.142(15)
β (°)	110.152(16)	110.027(12)	92.063(14)
γ (°)	92.33(2)	92.936(9)	93.937(14)
V (Å^3)	819.1(3)	843.69(18)	894.7(3)
Z	2	2	2
D_{calc} (Mg/m^3)	1.404	1.363	1.334
μ (mm^{-1})	0.104	0.101	0.098
F(000)	366	366	380
θrange (°)	2.69 - 27.50	2.67 - 27.50	2.34 - 27.50
Reflections collected / unique	10072/3722	8542/3831	12221/4017
[R(int)]	[0.0364]	[0.0344]	[0.0405]
Data/restraints/parameters	3722/0/238	3831/0/238	4017/0/256
Goodness-of-fit on F^2	1.070	1.033	1.020
Final*R1*, *wR2* indices [I>2*s*(I)]	0.0452, 0.1208	0.0503, 0.1328	0.0461, 0.1123
Final *R1*, *wR2* indices (all data)	0.0669, 0.1339	0.0909, 0.1581	0.0774, 0.1283
Largest diff. peak / hole (eA^{-3})	0.31/-0.28	0.27/-0.22	0.25/-0.23

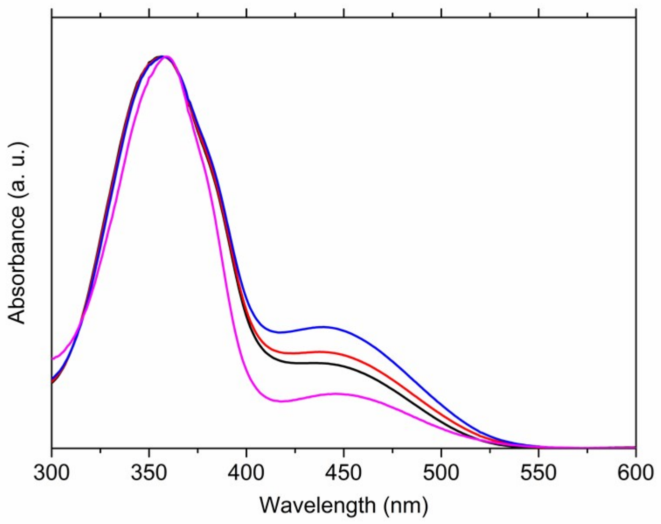

Fig. S4. Absorption spectra of **1** in DMF solution (magenta line) and thin film of **PAA-1$_{0.5}$** (blue line) **PAA-1$_{0.75}$** (red line) and **PAA-1$_{1.0}$** (black line).

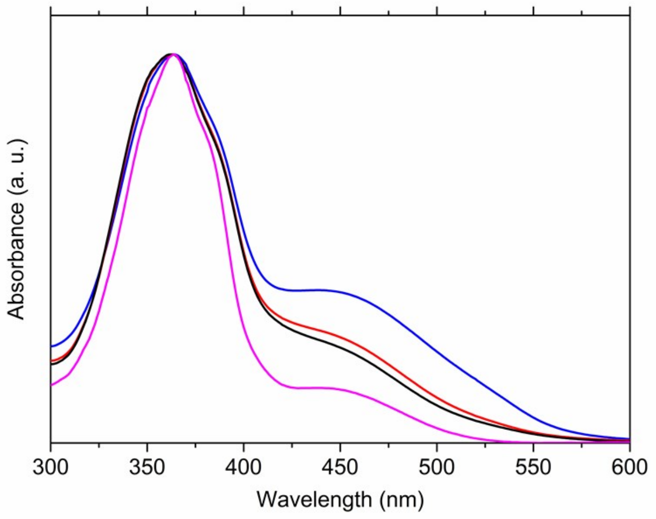

Fig. S5 Absorption spectra of **2** in DMF solution (magenta line) and thin film of **PAA-2$_{0.5}$** (blue line) **PAA-2$_{0.75}$** (red line) and **PAA-2$_{1.0}$** (black line).

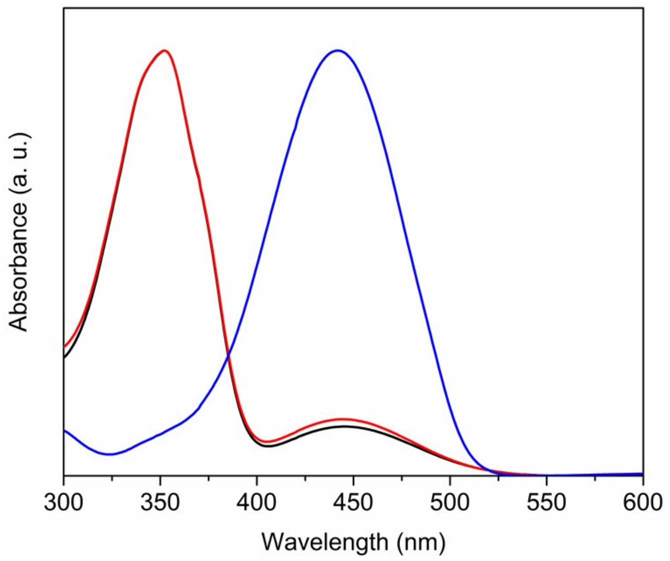

Fig. S6. Absorption spectrum of **1** in ethyl acetate (black line), ethyl acetate containing a small amount of acetic acid (red line) and acetic acid (blue line).

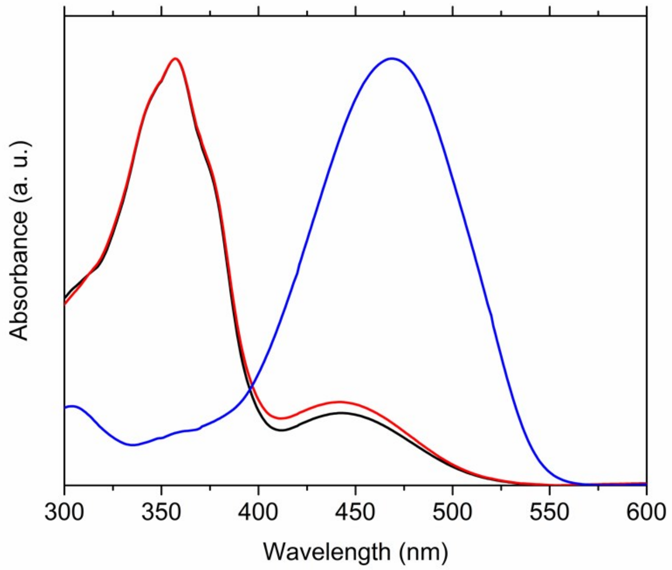

Fig. S7. Absorption spectrum of **2** in ethyl acetate (black line), ethyl acetate containing a small amount of acetic acid (red line) and acetic acid (blue line).

Tab. S3   Relevant data for the polymers.

	**PAA-1$_x$**			**PAA-2$_x$**			**PAA-3$_x$**
	x = 0.5	x = 0.75	x = 1.0	x = 0.5	x = 0.75	x = 1.0	x = 1.0
λ_{max} (nm)	357	357	357	364	364	364	343
n @633 nm	1.668	1.679	1.687	1.684	1.698	1.706	1.638
Modulation depth (nm)	190	245	420	110	273	355	40
Diffraction Efficiency (%)	6.65	14.5	30.9	6.42	15.0	23.5	1.94

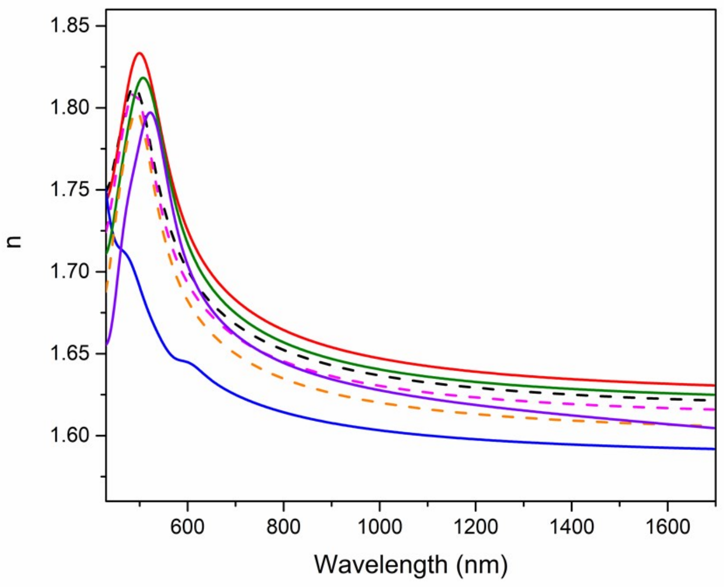

Fig. S8   Refractive index dispersion of **PAA-1$_x$** (orange, magenta and black dashed line for x = 0.5, 0.75, 1.0 respectively), **PAA-2$_x$** (purple, green and red solid line for x = 0.5, 0.75, 1.0 respectively) and **PAA-3$_{1.0}$** (blue solid line).

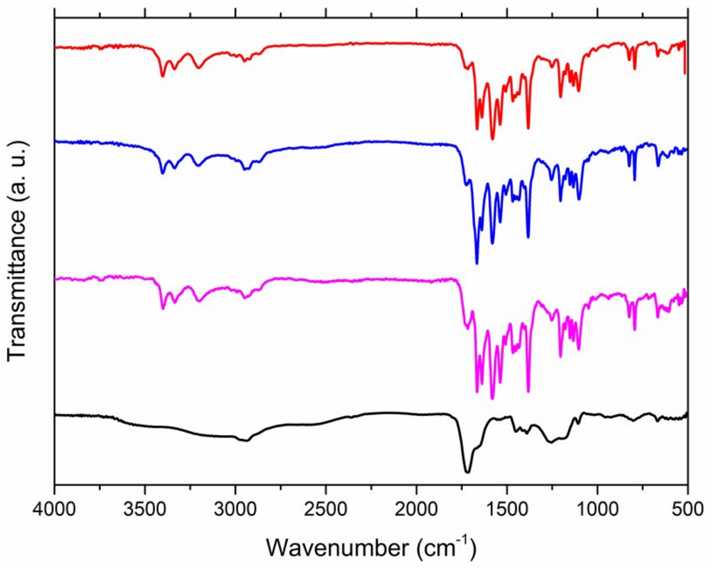

Fig. S9 FT-IR spectra of **PAA** (black line), **PAA-1$_{0.5}$** (red line) **PAA-1$_{0.75}$** (blue line) and **PAA-1$_{1.0}$** (magenta line).

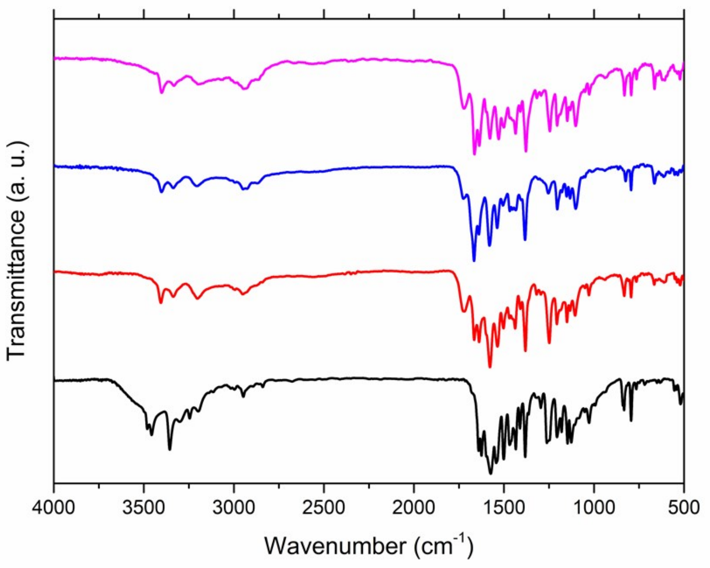

Fig. S10 FT-IR spectra of **2** (black line), **PAA-2$_{0.5}$** (red line) **PAA-2$_{0.75}$** (blue line) and **PAA-2$_{1.0}$** (magenta line).

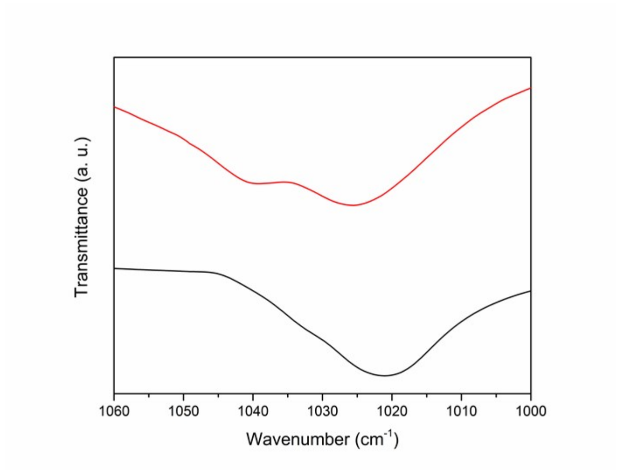

Fig. S11 FTIR spectra of 3 (black line) and **PAA-3**$_{\mathbf{1.0}}$ in the wavenumber region 1060-1000 cm^{-1}.

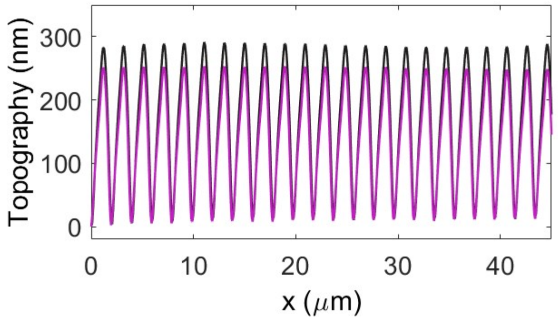

Fig. S12 AFM cross-sectional profiles of SRGs inscribed on **PAA-1**$_{\mathbf{0.75}}$ (grey line) and **PAA-2**$_{\mathbf{0.75}}$ (purple line) in the same time lapse.

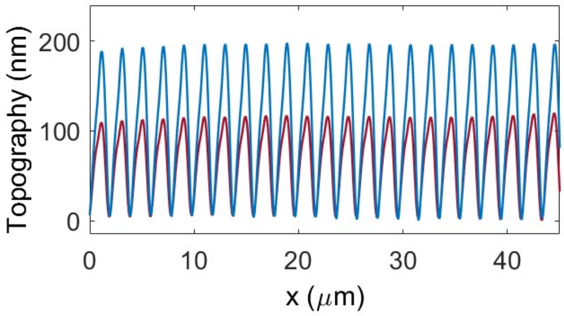

Fig. S13 AFM cross-sectional profiles of SRGs inscribed on **PAA-1**$_{\mathbf{0.50}}$ (blue line) and **PAA-2**$_{\mathbf{0.50}}$ (red line) in the same time lapse.

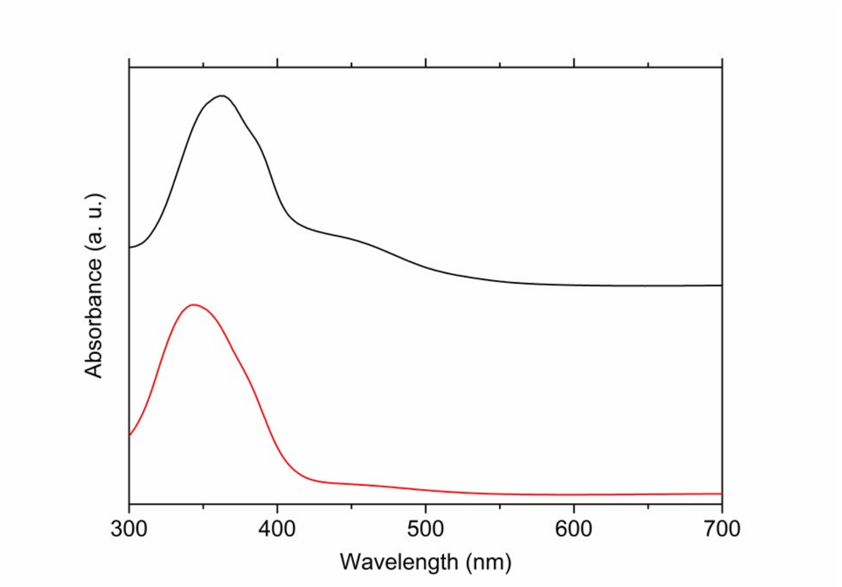

Fig. S14 Absorption spectra of **PAA-2$_{1.0}$** (black line) and **PAA-3$_{1.0}$** (red line) as thin films.

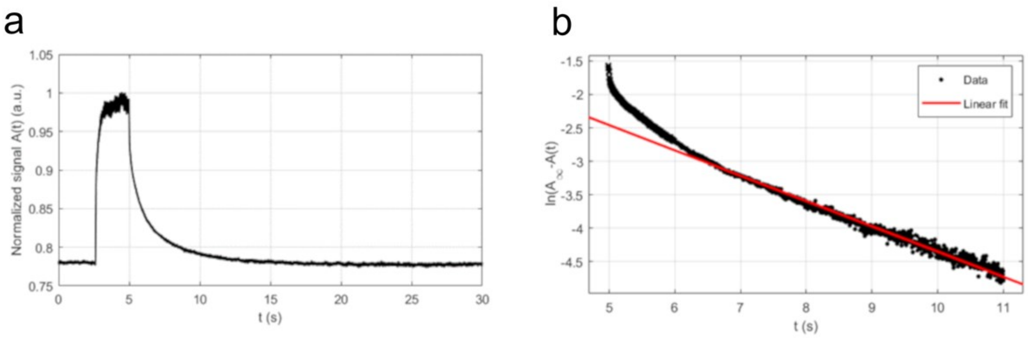

Fig. S15 Pump/relax curve (a) and plot of $ln(A_\infty - A(t))$ vs *t* (b) for the determination of *cis-trans* thermal isomerization rate *k* for **PAA-1$_{1.0}$**, **PAA-2$_{1.0}$** and **PAA-3$_{1.0}$**. A_∞ is the signal average values at long times (t > 20 s).

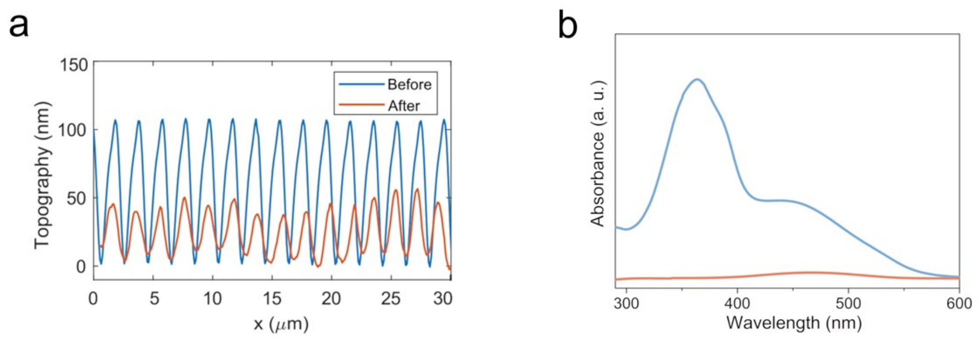

Fig. S16 Cross-sectional AFM profiles (a) and UV-vis spectra (b) of **PAA-2$_{0.50}$** film before (blue line) and after (orange line) rinse with solvent.
